# Dual Loading of Trans-Cinnamaldehyde and Either Paclitaxel or Curcumin in Chitosan Nanoparticles: Physicochemical Characterization and Biological Evaluation Against MDCK and HeLa Cells

**DOI:** 10.3390/polym16213087

**Published:** 2024-10-31

**Authors:** Cynthia L. Barrera-Martínez, Héctor I. Meléndez-Ortiz, Felipe Padilla-Vaca, Leonard I. Atanase, René D. Peralta-Rodríguez, Ioannis Liakos

**Affiliations:** 1 Centro de Investigación en Química Aplicada (CIQA), Blvd. Enrique Reyna Hermosillo 140, Colonia San José de los Cerritos, Saltillo C.P. 25294, Mexico; cynthia_barrera@uadec.edu.mx; 2 Consejo Nacional de Ciencia y Tecnología (CONAHCyT) Investigadoras e Investigadores por México, Commissioned at CIQA, Saltillo C.P. 25294, Mexico; 3 Departamento de Biología, División de Ciencias Naturales y Exactas, Universidad de Guanajuato, Noria Alta s/n Zona Universitaria, Guanajuato C.P. 36050, Mexico; padillaf@ugto.mx; 4 Faculty of Medicine, “Apollonia” University of Iasi, Pacurari Street, No. 11, 700511 Iasi, Romania; 5 Academy of Romanian Scientists, 050045 Bucharest, Romania; 6 Center for Micro-BioRobotics, Istituto Italiano di Tecnologia (IIT), Viale Rinaldo Piaggio 34, 56025 Pontedera, Italy; i.liakos@inn.demokritos.gr

**Keywords:** chitosan nanoparticles, curcumin, paclitaxel, trans-cinnamaldehyde

## Abstract

Biopolymer chitosan sub-micron particles (CSMPs) were prepared by the ionic gelation technique crosslinked with sodium tripolyphosphate co-loaded with trans-cinnamaldehyde (TCIN), and either curcumin (CUR) or paclitaxel (PTX). The size of the spherical CSMPs increased from 118 nm to 136 nm and 170 nm after the loading of TCIN and CUR, whereas the loading of PTX led to a slight decrease (114 nm). Polydispersity indexes of all the samples were smaller than 0.4, indicating monodisperse particles. Zeta potential values higher than +40 mV were determined, which is direct proof of the high stability of these nanoparticles. TCIN and PTX release studies in vitro, at pH 6.5 and 7.4, showed a pH dependence on the release rate with a higher value at pH 6.5. However, CUR was not released from CSMPs probably due to strong interactions with CS biopolymer chains. Cytotoxicity studies showed that the systems loaded with TCIN and PTX were more cytotoxic for HeLa cancer cells than for MDCK cells. Moreover, a synergistic effect against HeLa cells was observed for the TCIN-PTX-loaded CSMP samples. The Sensitivity Index indicated that the CSMPs loaded with TCIN have a prospective attraction to carry and release conventional or new chemotherapeutic drugs. This study demonstrates the in vitro efficiency of the obtained drug delivery system, but in vivo studies are necessary to confirm its potential for clinical applications.

## 1. Introduction

Nowadays, chemotherapy, surgery, and radiation therapy remain the main treatments for many types of cancer [1]. Particularly, chemotherapy has been highly effective against different forms of cancer, including testicular, lung, breast, and cervical [2]. However, almost all conventional chemotherapeutic drugs have drawbacks such as no targeting ability, short circulatory half-life in plasma, low water solubility, cancerous cell resistance, and serious side effects, mainly at high doses [3,4]. To avoid these drawbacks, polymeric nano (NPs) and micro-particles (MPs) have attracted considerable interest in the development of more effective anticancer drug delivery systems. Thus, they have become an important area of cancer nanotechnology investigation [5] due to the NPs’ high chemical and physical stability against severe environments, such as temperature, pH, and ultraviolet (UV) light [6,7]. These NPs can carry drugs, minimize side effects, increase circulation time, and improve therapeutic efficacy through targeted delivery of the encapsulated or entrapped drug [8,9].

NPs can be prepared from natural and synthetic polymers; however, the synthetic ones may be toxic for biomedical applications if they are not biocompatible and biodegradable [10]. On the contrary, NPs based on natural polymers are of greater interest due to their intrinsic properties, such as biocompatibility and biodegradability. Among the natural polymers, chitosan (CS) stands out as a prime research interest in drug delivery due to its unique characteristics, such as pH-responsiveness, biodegradability, and biocompatibility [11,12]. The biopolymer CS is a polysaccharide composed of glucosamine and N-acetylglucosamine units, containing large quantities of free amino groups that enable its crosslinking [13]. The physicochemical characteristics of CS, such as its low molecular weight (LMW) and a high degree of deacetylation (DD), have a major efficacy in tumor treatment since it generates high surface charge density NPs, resulting in the improvement of cellular uptake and antitumor efficacy. At neutral pH, CS is insoluble, but in an acidic solution, the NH_2_ groups become protonated (cationic nature; pKa ~ 6.5), making it water soluble and thus increasing cellular uptake by mucoadhesion with the negatively charged cell membrane [14,15,16,17]. The latter, due to its acidic pH sensitivity and similarity to the pH conditions of the tumor microenvironment, is, therefore, a suitable candidate for chemotherapeutic applications [16,17,18,19,20].

Paclitaxel (PTX) is one of the most common chemotherapeutic drugs, and curcumin (CUR) is a potential anticancer drug. PTX is one of the best-used anti-tumor drugs for various human malignancies because it promotes the assembly and stabilization of microtubules and interferes with essential cellular functions such as mitosis as well as cell transport and motility [21]. However, PTX has poor solubility in water due to its hydrophobic properties, which limits its clinical application. As for CUR, this is a bioactive hydrophobic polyphenol extracted from *Curcuma longa* and composed of two O-methylated phenols and one β–diketone, which has been widely used as a pharmaceutical or nutraceutical mainly due to its antioxidant, antiviral, anti-infection, and anti-inflammatory properties [11]. Also, extensive studies have shown the utility of CUR in cancer treatment as a chemopreventive, primarily because of its ability to arrest the cell cycle and induce tumor cell apoptosis without affecting normal cells [22,23]. However, the use of CUR has not been widely exploited for cancer treatment, mainly due to its extreme instability under physiological conditions and poor solubility in aqueous systems [23,24]. In addition, CUR faces additional problems, such as chemical and metabolic instability, which results in poor oral bioavailability [25].

The above limitations for both PTX and CUR, highlight the need to develop more effective delivery platforms with minimal side effects in cancer treatment. Such inconveniences can be overcome by applying several techniques, including chemical conjugation to obtain prodrugs [5,26], administration with metabolism inhibitors [27], and encapsulation in NPs [28,29,30]. In addition, for both drugs, the low aqueous solubility can be compensated using hydrophobic compounds, such as essential oils, as encapsulation precursors, which can, additionally, alleviate side effects. In this sense, essential oils, which are volatile, complex mixtures of compounds isolated from natural sources, have been used as pharmaceuticals to prevent and treat diseases [31]. Particularly, the cinnamon bark oil, whose main active ingredient (>80%) is *trans*-cinnamaldehyde (3-phenyl-2-propenal; TCIN), has been shown to possess antimicrobial properties and more recently, it has been reported to exhibit inhibition of the metabolic activities of cancer cells [32,33,34]. Therefore, TCIN enhances the solubility of drugs in the NPs and could have an effect on cancer cells. Further, TCIN has the advantages of leaving no residue after use, high safety, and low cost [35].

Loquercio et al. [36] prepared chitosan-alginate microparticles (MPs, >166 nm), which were loaded with TCIN by using the ionic gelation and polyelectrolyte complexation techniques. The antioxidant activity of TCIN-loaded MPs was studied with the aim of improving their effectiveness and efficiency for food delivery. Results showed that the scavenging ability of 2,2-diphenyl-1-picrylhydrazyl (DPPH) depended on the concentration of TCIN loaded into MPs. Joghataei et al. [37] encapsulated cinnamaldehyde into CS-based NPs with a size range of 60–80 nm to improve thermal stability and antioxidant activity for their application as nutraceutical and in the food industry. On the other hand, Liakos et al. [38] prepared NPs (140–165 nm) based on suberin, a biopolyester extracted from cork trees, and using the solvent/antisolvent method. These NPs were loaded with TCIN, and its synergistic effect against cancer cells was studied. They found that suberin nanoparticles containing TCIN loaded with 0.1% *w*/*w* PTX provided significant anticancer properties against human hepatocellular carcinoma HepG2 cancer cell lines. Also, they found that the single presence of TCIN and PTX did not give the suberin NPs’ anticancer activity. More recently, Zhu et al. [4] co-loaded docetaxel (7.82%) and CUR (6.48%) into MPs (>120 nm) of carboxymethyl chitosan, which showed a better synergistic anti-tumor effect in both in vivo and in vitro experiments than other nanocarriers loaded with DTX and CUR alone for the treatment of lung cancer. Although this study has confirmed the synergistic anticancer effect of CUR and docetaxel, there have been no further reports on the synergistic anticancer effect of CUR/PTX solubilized in the main component of the cinnamon bark oil.

Considering the above highlights, this work aimed to prepare CSMPs loaded with TCIN and PTX/CUR using the ionic gelation method to obtain new and more effective drug delivery systems. In as much as TCIN, PTX, and CUR possess anticancer properties, an attempt has also been made to explore their anticancer behavior in pH-responsive carriers, which is also a distinctive feature of the present work. The pH-responsive biopolymer chitosan can control the release of the bioactive agents by changing release conditions (e.g., pH) to increase the bioavailability of drugs. Additionally, another novelty feature of the present project lies in the fact that most of the reports on chitosan-based drug carriers encapsulate the active ingredients after particle preparation; in contrast, our group used an “in situ” encapsulation method. CSMPs loaded with TCIN and PTX/CUR were characterized by dynamic light scattering (DLS) to determine their particle size and zeta potential (ζp). In addition, the encapsulation efficiency (EE), TCIN, CUR, and PTX in vitro release, and cytotoxicity studies on both cancerous (HeLa) and non-cancerous (MDCK) cell lines were evaluated. It was expected that the loading of TCIN containing either PTX or CUR into CSMPs would provide a route to improve the anticancer activity via the synergistic effects of these compounds. Therefore, this research provides additional insights about essential oils’ synergistic activities when combined with anticancer drugs to obtain materials with improved anticancer activity and reduced toxicity.

## 2. Materials and Methods

### 2.1. Materials

Low molecular weight chitosan (50–190 kDa), *trans*-cinnamaldehyde (TCIN) (≥99%), curcumin (CUR) (≥94%), and in vitro toxicology assay kit MTT ((3-[4,5-dimethylthiazol-2-yl]-2,5-diphenyl tetrazolium bromide), sodium tripolyphosphate pentabasic (Na_5_P_3_O_10_) (≥98%) were purchased from Sigma-Aldrich (Saint Louis, MO, USA). Paclitaxel (PTX, ≥99%) was obtained from Selleck Chemicals LLC (Houston, TX, USA). Glacial acetic acid, sodium phosphate monobasic monohydrate (NaH_2_PO_4_·H_2_O), and sodium phosphate dibasic heptahydrate (Na_2_HPO_4_·7H_2_O) were obtained from Fermont (Monterrey, Mexico) and HPLC grade methanol was purchased from J.T. Baker (Phillipsburg, NJ, USA). In all the experiments, water from Milli Q equipment was used. HeLa, human cervical adenocarcinoma (ATCC CCL-2), and MDCK, Madin–Darby Canine Kidney (ATCC CCL-34) cells were maintained in Dulbecco’s modified Eagle medium (DMEM, Gibco, Grand Island, NE, USA) supplemented with 10% fetal bovine serum (FBS, Sigma-Aldrich).

### 2.2. Methods

#### 2.2.1. Preparation of TCIN and CUR/PTX Co-Loaded CSMPs

Non-loaded CSMPs were prepared according to the previously reported methodology [39]. First, a CS solution (1 mg/mL) was prepared in a 1% acetic acid solution. Then, 24 mL of this solution was placed in a jacketed glass reactor, mechanically stirred (450 rpm), and heated at 60 °C for 15 min. Then, 5 mL of a TPP aqueous solution (2 mg/mL) was added to this solution, and the resulting mixture was allowed to react for 30 min.

TCIN-loaded CSMPs were prepared similarly with some modifications. First, TCIN (48 mg) was added to the CS solution and stirred for 15 min before the addition of the TPP aqueous solution. Regarding the CUR and PTX loading processes, either CUR or PTX (20 mg) was first dissolved in 1 mL of TCIN. Subsequently, the corresponding mixture of TCIN/drug (48 mg) was placed in a jacketed glass reactor, and 24 mL of CS solution was added. This mixture was allowed to react for 30 min. Afterward, 5 mL of a TPP aqueous solution (2 mg/mL) was added to the mixture and stirred for 30 min. All procedures were carried out at room temperature, and the final mixtures were allowed to stand overnight. Subsequently, the reaction mixtures were frozen and lyophilized (−40 °C, 0.3–0.5 mBar for 48 h; Labconco FreeZone1, Kansas City, MO, USA) for further characterization. The values of particle diameter, Dp, and zeta potential, ζ_p_, of both non-loaded and drug-loaded CSMPs were determined by dynamic light scattering (DLS) in a Microtrac Nanotrac Wave II and Microtrac ZETA-check Particle Charge Reader (Montgomeryville, PA, USA), respectively. The morphology of particles was analyzed by scanning electron microscopy (JEOL JSM-7401F, Tokyo, Japan; and Dual Beam equipment, Sarasota, FL, USA). All experiments were carried out in duplicate.

#### 2.2.2. Determination of the Entrapment Efficiency (EE)

A lyophilized drug-loaded CSMP sample (5 mg) was dispersed in 1 mL of methanol and centrifuged at 13,500 rpm for 5 min at 4 °C (Select BioProducts, SelectSpin 17R microcentrifuge). Then, the supernatant was collected to quantify the entrapped amount of TCIN, CUR, and PTX by UV spectrophotometry at the wavelengths of 285, 420, and 230 nm, respectively (Thermo Scientific, Multiskan GO, Waltham, MO, USA). Previously, calibration curves in methanol were constructed for each component (TCIN (R^2^ = 0.995); CUR (R^2^ = 0.998); PTX (R^2^ = 0.990)). These experiments were performed in duplicate. The entrapment efficiency (EE) was calculated using Equation (1):

(1) $$EE\left(\%\right)=\frac{Amount\;of\;drug\;within\;CSMPs}{Total\;amount\;of\;drug}\times 100$$

#### 2.2.3. In Vitro CUR and PTX Release Studies

A sample (2 mg) of the corresponding drug-loaded lyophilized CSMP was dispersed in 1 mL of the corresponding PBS solution (pH 7.4 or 6.5). The samples were kept in an incubator (Benchmark, Incu-Shaker 10 L, Tempe, AZ, USA) at 37 °C and 200 rpm. At given times, the tubes were centrifuged at 13,500 rpm for 10 min, and the supernatants were withdrawn and collected. Then, the tubes with the sample were filled with 1 mL of fresh PBS solution and returned to the incubator to continue the release study. The collected supernatant (PBS solution) was dissolved in methanol (1:1 *v*/*v*), and the amount of TCIN, CUR, and PTX was quantified spectrophotometrically at the corresponding wavelength. The amount of each compound was obtained from the respective calibration curves at different pH values.

Then, the remaining sediments were dispersed by adding 1 mL of methanol and stirred overnight. Afterward, these dispersions were sonicated using a probe sonicator (Fisher Scientific, 700 W model FB705, Pittsburgh, PA, USA) at 5% amplitude for 1 min. Then, the dispersions were centrifuged at 13,500 rpm for 10 min at 4 °C, and the amount of TCIN, CUR, and PTX were quantified in the supernatant. The R^2^ values for the calibration curves of TCIN, CUR, and PTX at pH 7.4 were 0.997, 0.983, and 0.988, respectively, while at pH 6.5 were 0.993, 0.995, and 0.960. These experiments were performed in triplicate, and the drug released was calculated from Equation (2):

(2) $$Release\;\left(\%\right)=\frac{Released\;drug\;from\;CSNPs}{Total\;drug\;entrapped\;in\;CSNPs}\times 100$$

#### 2.2.4. Cytotoxicity Assay

The cytotoxic activity of free CUR and PTX (dissolved in DMSO), TCIN, TCIN-CUR, and TCIN-PTX loaded CSMP samples were evaluated in both cancerous (HeLa) and non-cancerous (MDCK) cell lines. HeLa and MDCK cells were seeded into 96-well plates at a density of 25,000 and 15,000 cells/well, respectively. The cells were incubated in DMEM supplemented with 10% FBS at 37 °C with 5% CO_2_ for 24 h. Then, in a biosafety cabinet, loaded CSMPs at determined concentrations (TCIN (0–50 μM); CUR (0–20 μM); PTX (0–50 μM)) were added to the wells and incubated at 37 °C with 5% CO_2_ for 24 h. Cell viability was measured following the MTT method as previously described [35]. Briefly, after incubation, the medium was removed, and 90 μL of PBS solution was added to each well, followed by 10 μL of MTT (5 mg/mL) solution. Then, samples were incubated for 2 h at 37 °C and 5% CO_2_. The insoluble formazan produced was solubilized in 100 μL of ethanol, and the optical density was measured at 570 nm in a microplate reader (Multiskan GO, Thermo Scientific). The cell viability was calculated, and the data were expressed as the average of at least three independent experiments. The IC_50_ values of free CUR and PTX (dissolved in DMSO), TCIN, TCIN-CUR, and TCIN-PTX-loaded CSMPs were determined by linear regression calculations from the concentration and inhibition percentage curve using at least four concentrations (data obtained by the MTT assay method). The significance of straight line fit was determined using critical values for Pearson’s correlation coefficient considering 5% significance. Additionally, the selectivity index (SI) used as an indicator of the anticancer activity of a sample, was determined. SI values were calculated by Equation (3) [40]:

(3) $$SI=\frac{{IC}_{50}\;non\;cancer\;cells}{{IC}_{50}\;cancer\;cells}$$

#### 2.2.5. Statistical Analysis

The experimental data reported here are the average results of two or more determinations, and the error is shown as the standard deviation for each case.

## 3. Results and Discussion

### 3.1. Preparation and Characterization of TCIN and CUR or PTX Co-Loaded CSMPs

The non-loaded and drug-loaded CSMPs were characterized by DLS, and the Dp and ζ_p_ values are shown in Table 1.

**Figure 1:**
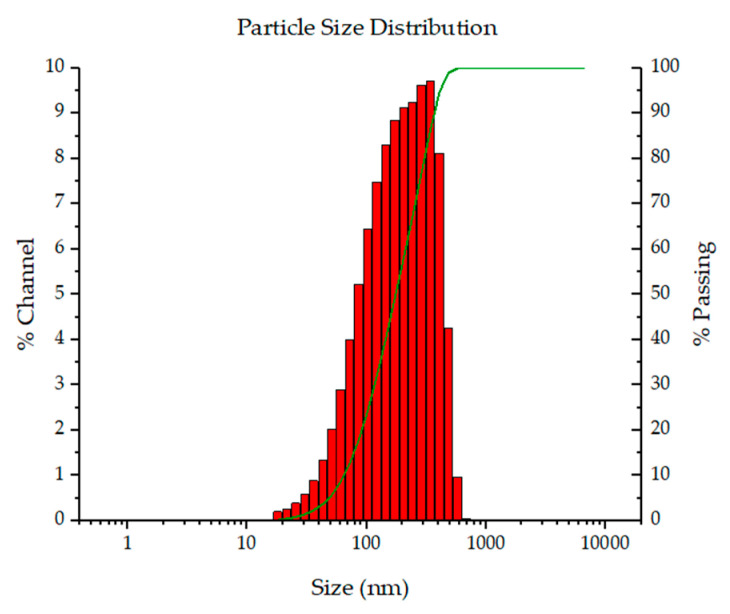
Particle size distribution of TCIN-loaded CSMPs.

The Dp values for the non-loaded CSMPs (118 nm) were smaller than those for the TCIN-loaded CSMPs (136 nm). Similarly, samples containing CUR or PTX showed Dp values of 170 and 114 nm, respectively. All systems showed polydispersity index (PDI) values ≤ 0.4, indicating monodisperse particle size distributions (PSD), as demonstrated by the representative image shown in Figure 1.

Li et al. prepared CS nanoparticles loaded with PTX by solvent evaporation and emulsification crosslinking methods with an average size of 116 nm, which are very similar to the ones determined for the TCIN-PTX-loaded CSMP samples [41]. Other authors have reported the loading of PTX into CS particles with an average size ranging from 140 to 270 nm using other preparation methods [42,43,44,45]. For the case of CUR, the loading of this compound into both CS and CS derivatives NPs with average sizes ranging from 190 to 230 nm has also been reported [4,8,22]. As mentioned in the literature, particles smaller than 10 nm are quickly eliminated by renal changes, while those larger than 300 nm could be removed from the blood circulation due to recognition by the reticuloendothelial system [46,47]. Therefore, the obtained sizes for the TCIN-loaded CSMPs with CUR or PTX in this work are ideal for their use as carriers of anticancer drugs.

**Figure 2:**
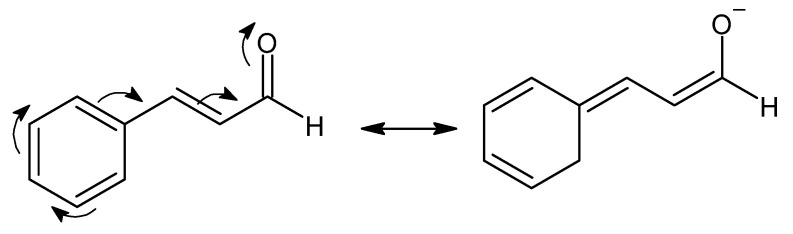
Resonance structure of trans-cinnamaldehyde. Created with ChemSketch.

With respect to the values of ζ_p_, no differences were found for the non-loaded and loaded samples. It is proposed that this result could be attributed to an encapsulation of the oil within the chitosan molecular network that forms the CSMPs [48]. By analyzing the resonance structures of TCIN (Figure 2), it can be observed that the oxygen of the carbonyl group possesses a negative charge that interacts with the positive charge of the CS, decreasing the net surface charge of the particles and, thus, lowering the value of ζ_p_. In fact, it can be assumed that some essential oil is adsorbed on the surface of the particles, masking the protonated free amino groups of CS chains. The positive values of ζ_p_ measured were due to the presence of protonated amino groups from the CS chains located on the outer-most surfaces of the NPs, demonstrating the TCIN, CUR, and PTX encapsulation efficiency within the CSMPs. These positive values indicate that the systems reported herein form physically stable suspensions as indicated for ζ_p_ values above +30 mV [49].

On the other hand, the values of EE (%) for the TCIN-CUR-loaded CSMPs were 7.4 and 23.6% for TCIN and CUR, respectively, while for the TCIN-PTX-loaded CSMPs, values of 7.9 and 100% were obtained for TCIN and PTX, respectively. The relative low EE of TCIN in the CSMPs is ascribed to the fact that the freeze-drying technique applied for each system causes a significant loss of the main component of cinnamon bark oil during drying, decreasing the amount of TCIN quantified by spectrophotometry [50].

The high PTX load in CSMPs can be one of the causes for their lower size (114 nm) compared to the CUR load due to PTX’s surface charge and/or surface tension properties and good solubility and retention of CSMPs that lower their size during NPs formation. The effective retention of PTX within CSMPs can lead to a more compact structure, resulting in smaller nanoparticle sizes. Furthermore, the high PTX load in CSMPs not only influences their size but also enhances their stability and prolongs the release profile of the drug, making them a promising drug delivery system with controlled and sustained drug release characteristics.

**Figure 3:**
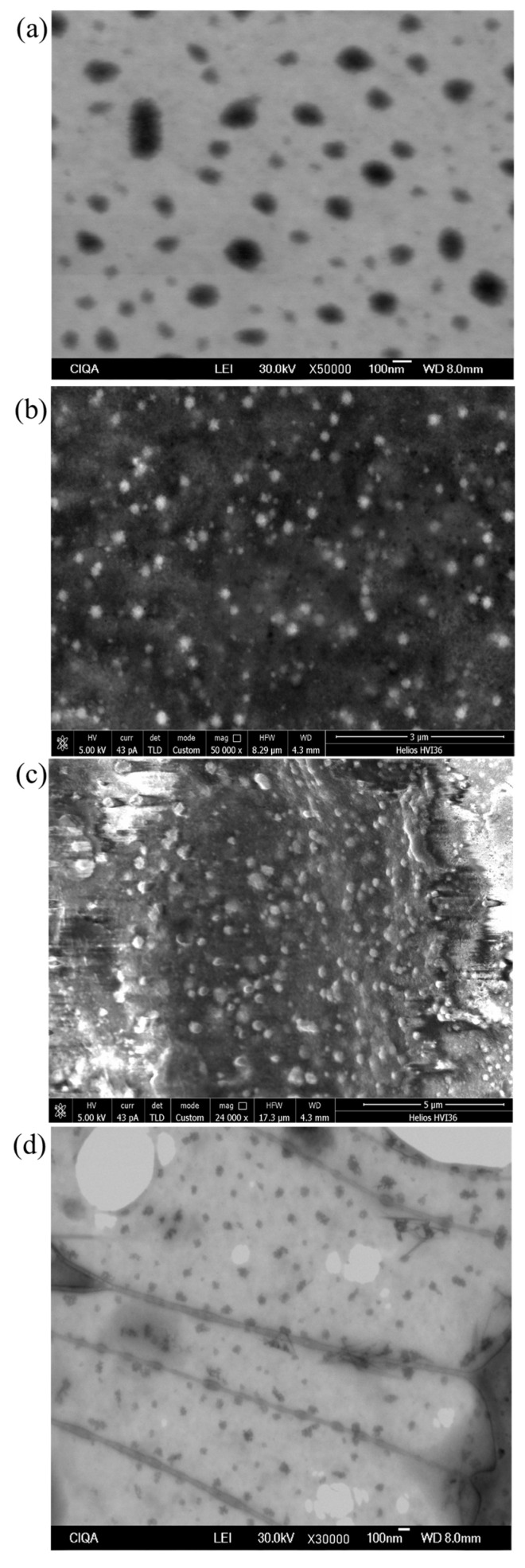
SEM micrographs of (**a**) non-loaded, (**b**) TCIN-loaded, (**c**) TCIN-CUR-loaded, and (**d**) TCIN-PTX-loaded CSMPs.

Further, particle morphology is one of the most important parameters in the characterization of polymeric NPs because their pharmaceutical application depends on it. Parameters like cellular uptake, cargo release, and drug clearance are usually affected by particles’ morphology and surface chemistry [51,52]. Usually, CSMPs have a unique morphology depending on the preparation method, crosslinking agent, and drying technique, among other factors. SEM micrographs of the non-loaded and loaded CSMPs showed a sponge-like spherical morphology (Figure 3).

This morphology is formed due to several factors. One is related to the CS: TPP ratio and pH of the reaction medium, which greatly influences the size and porosity of particles. Additionally, while samples are in the drying process, solvent evaporation promotes pore formation, and often, hydrogen bonding interactions become predominant, which could generate particle agglomeration [53,54].

### 3.2. In Vitro TCIN, CUR, and PTX Release Studies

**Figure 4:**
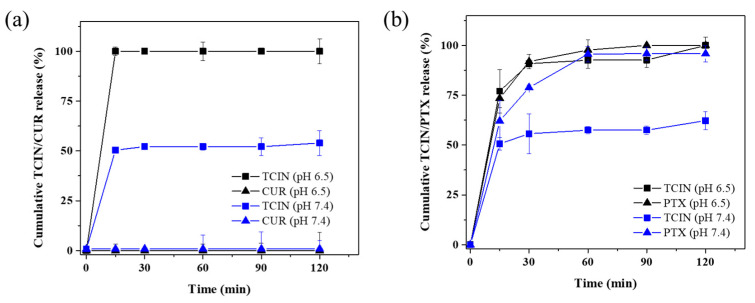
In vitro release tests at pH 6.5 (black symbols) and 7.4 (blue symbols). (**a**) TCIN-CUR-loaded and (**b**) TCIN-PTX-loaded CSMPs.

In vitro TCIN, CUR, and PTX-loaded CSMPs release studies were carried out in phosphate buffer solution (pH 7.4 and 6.5) under sink conditions (excess of release medium). Figure 4a shows the release profiles of TCIN-CUR-loaded CSMPs at different pH values.

It can be observed that a burst release of TCIN at both pH values is more evident at pH 6.5. This behavior was due to the fact that at pH 6.5, the chains of CS are ionized, causing repulsion between the protonated amino groups and facilitating the release of TCIN [42]. On the contrary, CUR was not detected in the release medium at both pH values. This phenomenon could be explained due to the hydrophobic nature of CUR. In contact with aqueous solutions, CUR tends to form crystals; these crystals entrapped in the CSMPs would hamper diffusion of the CUR from the chitosan matrix, resulting in an irregular release profile [55]. Furthermore, the negatively charged oxygen atom in the CUR chemical structure could create a bond with protonated amino groups in chitosan chains [56]. In addition, the interactions of the hydroxyl groups from CUR with the glucosamine units in the CS molecules could contribute to this behavior at pH 7.4 [57]. However, to demonstrate the presence of CUR captured in the CSMPs and probably attached to chitosan, the samples were sonicated (5% amplitude for 1 min) to break any possible interaction. Subsequently, samples were centrifuged to analyze the supernatant, which gave, as a result, a CUR release of 89.0 ± 3.1 and 62.1 ± 7.4% at pH 6.5 and 7.4, respectively.

On the other hand, for the TCIN-PTX-loaded CSMP samples (Figure 4b), more TCIN was released at pH 6.5 than at 7.4. TCIN showed a burst release with a cumulative percentage of 77% in the first 15 min at a pH of 6.5, reaching 100% after 120 min, while at a pH 7.4, an initial burst release of 53% was observed, reaching complete delivery after 240 min. The above results were in agreement with the intrinsic pH-dependence characteristic of CS.

Besides, the PTX release profile showed 74% and 65% values during the first 15 min at pH 6.5 and 7.4, respectively, reaching complete release at 120 min in both cases. The main mechanisms for the release of drugs from polymeric matrix systems in vitro are swelling, diffusion, and disintegration [58]. Since the grade of CS used in the present study was of low molecular weight with a degree of deacetylation ≥77.4%, an important fraction of this significant release after 15 min is attributed to these characteristics of CS [39]. In addition, a major diffusion of PTX is expected at pH 6.5 due to an increase in swelling by the CS chains. This behavior is explained because, under acidic conditions, the CS chains become protonated, causing the hydrogen bonds between the amino groups of CS and the unionized PTX molecules to break (pKa = 11.9) [59]. These results showed a burst release behavior for the encapsulated compounds in CSMPs. This phenomenon has been well documented in the specialized literature [60,61,62], and despite not being an ideal sustained release system, the results are important since the system showed a pH dependence. Hence, even when the compounds are released rapidly, this behavior could be useful for targeted delivery to tumors [63]. Previous works on the PTX release entrapped in CS polymeric matrices have been reported [41,45,58]. However, most of those works report very slow PTX release rates, reaching values close to 100% after several days. Frequently, it is desired that a rapid release happens in the first stage to treat the disease, followed by a slow-release stage to consolidate the curative effect [58]. Therefore, further efforts must be made to control the PTX release from nano and/or microcarriers to find a satisfactory delivery of this drug.

### 3.3. Cytotoxicity Assay

**Figure 5:**
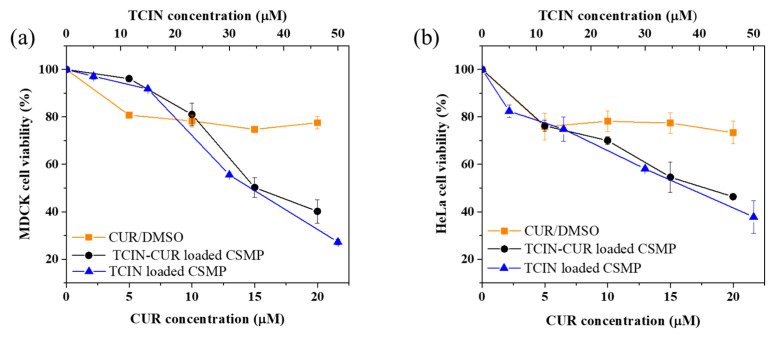
Cell viability of (**a**) MDCK and (**b**) HeLa cells exposed at 24 h for CUR/DMSO, TCIN-loaded CSMPs, and TCIN-CUR-loaded CSMPs.

The effects of the TCIN-loaded and TCIN/drug-loaded CSMPs on the viability of both MDCK and HeLa cells were evaluated using the MTT assay. In addition, the corresponding drug (CUR or PTX) solubilized in DMSO was also tested. IC_50_ is defined as the half-maximal inhibitory concentration and indicates the concentration of drug/compound necessary to inhibit 50% of either non-cancer or cancer cells. It is strongly dependent on the methodology used. Another indicative parameter for the potential of a drug system to treat diseases is the selectivity index (SI), defined as the ratio between the effective concentration and the toxic concentration of a sample. The SI is a strong guide to evaluate the anticancer activity of a sample and the cytotoxic selectivity for cancer cells relative to normal cells. Generally, SI ≤ 3 indicates cytotoxicity in normal cells, SI ≥ 3 is classified as a potential candidate anticancer sample, and SI ≥ 10 could be considered as a selected sample for further clinical investigation [40]. From Figure 5a,b, it can be observed that the CUR/DMSO sample showed low cytotoxicity in both MDCK and HeLa cell lines with values of cell viability between 73–78% in the concentration range tested, 10–20 µM for both MDCK and HeLa cells; that result could be correlated with similar IC_50_ values for both cell lines (Table 2).

On the other hand, the TCIN and TCIN-CUR-loaded CSMP samples (Figure 4) showed more cytotoxic effects on both cell lines than free CUR/DMSO.

Similar results have been previously reported where the free CUR is less toxic to HeLa cancer cells than the encapsulated drug [64,65]. The above-mentioned effect was also observed in IC_50_ values, where for the HeLa cell line, a higher value was obtained for free CUR (39.94) than for the CUR-entrapped system (17.54). Kumari et al. [66] reported similar results: higher efficiency was observed for its nano-formulation systems rather than for free CUR on breast cancer cells.

The viability of MDCK cells was reduced at TCIN concentrations above 15 μM, while for the HeLa cells, a concentration-dependent behavior of cell viability was observed as the concentration of TCIN was increased for both samples.

Furthermore, no significant differences were found in the cytotoxicity of the TCIN and TCIN-CUR-loaded samples for both cell lines, in agreement with previous CUR delivery studies where it was observed that CUR was not released from CS matrices; therefore, it could not contribute significantly to cytotoxicity.

**Figure 6:**
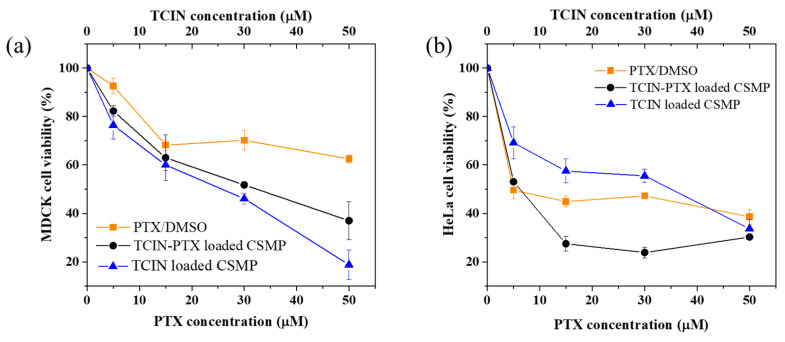
Cell viability of (**a**) MDCK and (**b**) HeLa cell lines exposed during 24 h for the PTX/DMSO, TCIN-loaded CSMPs, and TCIN-PTX-loaded CSMPs.

Regarding samples loaded with TCIN and PTX, the PTX/DMSO sample showed greater cytotoxicity against HeLa cells (cell viability 39%) than MDCK cells (cell viability 62%) at a concentration of 50 µM (Figure 6a,b).

This behavior agrees with the obtained IC_50_ values, where it should be noted that the HeLa cell line has a more responsive effect on PTX (17.38) than the MDCK cell line (61.02). In addition, progressive cytotoxicity was observed for the MDCK cells as the PTX concentration increased, while for HeLa cells, the cytotoxicity could be observed even at relatively low concentrations. Some authors have reported the cytotoxicity of PTX on several cell lines, including DU-145 prostate cancer, G361 melanoma cancer, MDA-MB-231 breast cancer, and A2780 ovarian cancer [41,43,44,45,58]. In most of these reports, the incorporation of PTX into CS nanoparticles increased the cytotoxicity of this drug.

On the other hand, it was observed that the CSMP sample loaded with TCIN and PTX showed the greatest cytotoxic effect on HeLa cells, reducing the cell viability until approximately 28% at low PTX and TCIN concentrations of 15 µM. These results suggest a synergistic effect between TCIN and PTX against HeLa cancer cells since the TCIN-loaded CSMPs showed lower cytotoxicity at the same concentration (cell viability of 57%). Also, it can be observed that the TCIN-PTX-loaded CSMPs system showed lower cytotoxicity against MDCK lines when compared with the HeLa cells, which could be an advantage for the development of drug delivery systems of anticancer agents.

**Figure 7:**
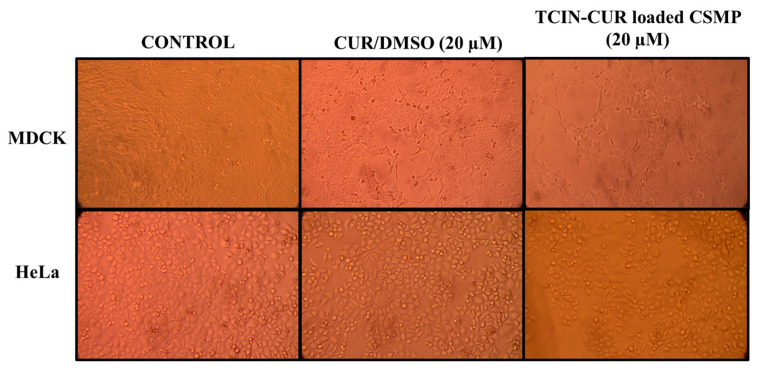
Light microscopy images of MDCK and HeLa cell lines exposed to CUR/DMSO and TCIN-CUR-loaded CSMPs for 24 h. Image taken with digital camera of 40× and 100× objectives.

The exposure of MDCK and HeLa cells to CUR, PTX, and the TCIN-CUR and TCIN-PTX-loaded CSMP samples induced cell damage and destruction of the cell monolayer for both cell lines, as shown in Figure 6 and Figure 7. Figure 7 shows that the MDCK and HeLa monolayer cells without drug remained intact after 24 h of incubation.

Cell monolayers treated with the CUR/DMSO showed few gaps because the cell monolayer did not reach 100% confluence due to a little growth inhibitory effect either by CUR/DMSO or TCIN-CUR-loaded CSMPs. For both HeLa and MDCK cell lines, the SI value (1.04) showed that CUR has no cell selectivity and is capable of equally affecting both monolayers. On the other hand, the cells incubated with TCIN-CUR-loaded CSMPs showed a higher number of detached cells, in agreement with what is shown in Figure 5 and with lower IC_50_ values (Table 2); the same behavior has been reported recently for a chitosan encapsulated system [67].

**Figure 8:**
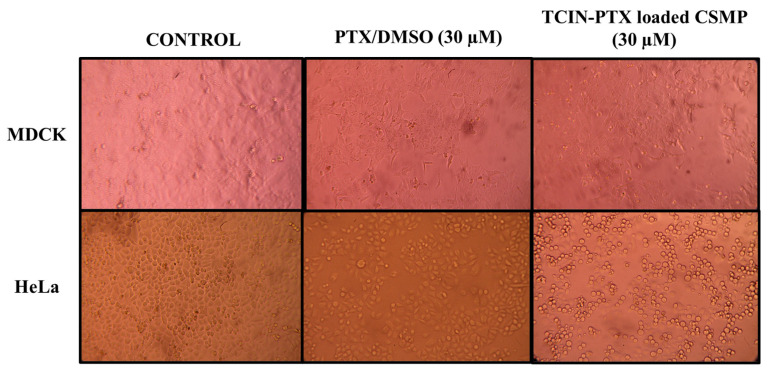
Light microscopy images of MDCK and HeLa cell lines exposed to PTX/DMSO and TCIN-PTX-loaded CSMPs for 24 h. Image taken with digital camera of 40× and 100× objectives.

Figure 8 shows that TCIN-PTX-loaded CSMPs have a greater effect on HeLa cells than MDCK cells, where completely rounded and detached HeLa cells were observed, which could be confirmed by its SI value ≥ 3 (5.87). The TCIN and PTX released from the CSMPs favored the cancer cells. These results are correlated with the previously discussed cytotoxic studies, indicating that when the particles were loaded with the main component of the cinnamon bark oil and PTX, they showed a greater effect than the free drug.

**Figure 9:**
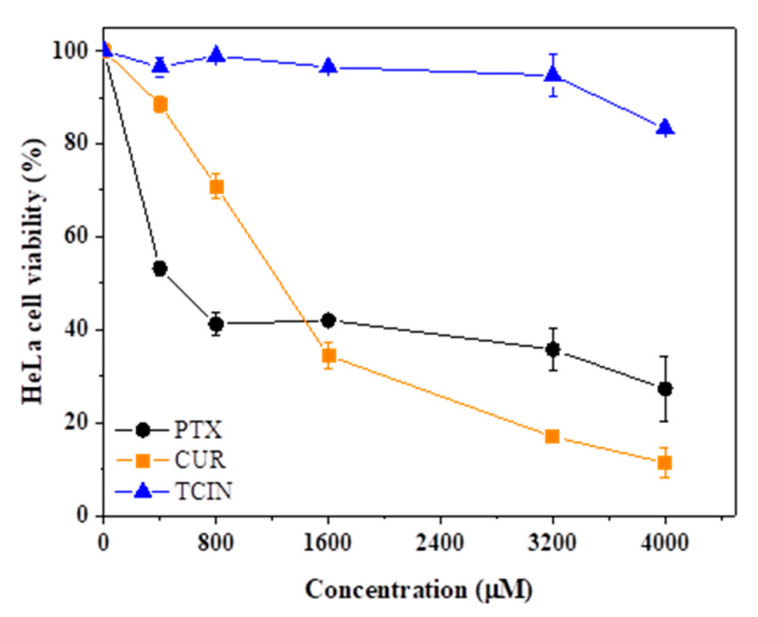
Viability (%) of HeLa cells exposed for 24 h to PTX, CUR, and TCIN in DMSO at concentrations from 400 to 4000 µM.

Finally, to explain the effect on the cell viability of free drugs plainly, it must be considered that these compounds are homogeneously solubilized in DMSO, while the entrapped drugs tend to sediment at the bottom of the wells. The cytotoxicity behavior can be explained by the fact that the incorporated drugs are in direct contact with cells, favoring their interaction and, therefore, the cytotoxic effect. Considering the compounds loaded into CSMPs (TCIN, CUR, and PTX), each one was tested separately to study their cytotoxic effect on HeLa cancer cells, and the results are shown in Figure 9.

In HeLa cells, PTX exhibited more cytotoxicity at low concentrations (<800 μM) compared to TCIN and CUR, while CUR exhibited a greater effect than TCIN and PTX, but only at high concentrations (>1600 µM), whereas TCIN showed the lowest effect in comparison with CUR and PTX.

## 4. Conclusions

Spherical nanoparticles CSMPs were prepared and loaded with *trans*-cinnamaldehyde (the main component of the cinnamon bark oil), CUR, and PTX for testing as anticancer agent delivery systems. The TCIN was co-loaded with either CUR or PTX into CSMPs to minimize the problems associated with drug delivery, including poor solubility, bioavailability, and degradation. CSMPs were able to entrap up to 9% of TCIN while the EE of CUR and PTX was 26 and 100%, respectively, demonstrating that TCIN, other than its bioactivity, can be a good precursor for encapsulating water-insoluble CUR and PTX into CSMPs. It was found that the cumulative release of TCIN and PTX was higher at tumor microenvironment pH (6.5: 77% TCIN; 74% PTX)) than at neutral pH (7.4: 53% TCIN; 65% PTX). However, CUR was not released. The prepared drug delivery systems exhibited a burst release in the first minutes and a gradual release rate thereafter, which may be positive in treating tumors and should be considered when evaluating their performance in vivo in other studies. The TCIN and drug-loaded CSMP systems showed a higher cytotoxic effect against cancer cells when compared with the corresponding free drugs. The PTX-loaded samples showed 26% higher cytotoxicity against cancer cells than the CUR-loaded ones. In addition, a synergistic effect was found for the TCIN-PTX-loaded CSMPs sample against the HeLa cell line. The TCIN-PTX-loaded CSMPs with an SI value ≥ 3 (5.87) have the potential to be optimized to develop a new commercial anticancer treatment system. The results obtained in this work demonstrated that the co-loading of the main components of essential oils and bioactive anticancer agents into CSMPs could be promising potential drug delivery carriers for cancer treatment.

## Figures and Tables

**Table 1:** Dp, PDI, ζ_p_, and EE values for the non-loaded and TCIN/drug-loaded CSMP samples.

Sample	D_p_ (nm)	PDI	ζ_p_ (mV)	EE (%)	
				TCIN	Drug
Non-loaded CSMPs	118.0 ± 0.4	0.20 ± 0.01	+43 ± 0.6	---	---
TCIN-loaded CSMPs	136.0 ± 1.0	0.30 ± 0.03	+42 ± 2.0	9.1 ± 2.0	---
TCIN-CUR-loaded CSMPs	170.0 ± 8.1	0.40 ± 0.01	+40 ± 2.0	7.4 ± 3.0	23.6 ± 6.4
TCIN-PTX-loaded CSMPs	114.0 ± 1.3	0.30 ± 0.01	+42 ± 1.6	7.9 ± 0.1	100 ± 3.5

**Table 2:** IC_50_ and SI values for the non-loaded and TCIN/drug-loaded CSMP samples.

Sample	IC_50_ (μM)		SI
	MDCK	HeLa	
CUR/DMSO	41.71 ± 3.81	39.94 ± 5.66	1.04
PTX/DMSO	61.02 ± 4.49	17.38 ± 0.02	3.51
TCIN-loaded CSMPs	26.97 ± 1.00	16.67 ± 1.48	1.61
TCIN-CUR-loaded CSMPs	17.10 ± 0.16	17.54 ± 0.61	0.97
TCIN-PTX-loaded CSMPs	34.43 ± 3.02	5.86 ± 0.92	5.87

## Data Availability

Data is available upon request.
